# Developing an Empirical Theory of Planned Behavior Model of Healthy Dietary Choice and Evaluating Gamified Feedback Among Japanese Young Adults

**DOI:** 10.3390/nu18040686

**Published:** 2026-02-20

**Authors:** Yutaka Akitsu, Yoko Yamakata, Eiji Yamasue

**Affiliations:** 1 Research Organization of Science and Technology, Ritsumeikan University, 1-1-1 Nojihigashi, Kusatsu City 525-8577, Shiga, Japan; 2 Interdisciplinary Information Science Research Division, Information Technology Center, The University of Tokyo, 7-3-1 Hongo, Bunkyo-ku 113-8654, Tokyo, Japan; yamakata@hal.t.u-tokyo.ac.jp; 3 Faculty of Science and Engineering, Ritsumeikan University, 1-1-1 Nojihigashi, Kusatsu City 525-8577, Shiga, Japan; yamasue@fc.ritsumei.ac.jp

**Keywords:** Theory of Planned Behavior, dietary behavior, self-efficacy, gamification, young adults, mobile nutrition app, FoodLog Athl

## Abstract

Background/Objectives: Dietary behaviors among young adults in Japan have become increasingly polarized, highlighting the limitations of traditional knowledge-based health education. Behavioral science-based approaches such as nudging and gamification may offer alternative strategies. This study aimed to develop and examine a Theory of Planned Behavior (TPB)-based path model of healthy dietary choice behavior among young Japanese adults and to examine patterns associated with a star-rating gamification feature embedded in a nutrition management mobile application. Methods: A total of 188 participants aged 18–39 years completed an online survey assessing TPB constructs and normative factors. Participants used either a star-rating or non-rating version of the FoodLog Athl application. Composite-score-based path analysis and conditional process analyses were conducted to examine relational patterns among constructs. Results: Intention and self-efficacy jointly explained 48% of the variance in dietary behavior, with self-efficacy emerging as the strongest predictor. Several moderation patterns were observed, including those of gender, university year, diet app use, awareness of consequences, and ascription of responsibility. Compared with users of the non-rating version, star-rating users were observed to show higher nutrient scores but lower self-efficacy and dietary behavior scores, along with greater awareness of dietary consequences. These post-intervention findings are exploratory. Conclusions: Self-efficacy plays a central role in healthy dietary choice behavior among young adults, and its association with behavior appears to be shaped by perceived consequences and responsibility. By applying a composite-score-based path analysis within an SEM framework, this study clarifies the structural relationships among TPB components in everyday dietary choice behavior among Japanese young adults. Star-rating feedback may enhance reflective awareness and shows potential as a gamified nudging tool but further research is needed to clarify its effects.

## 1. Introduction

Japan’s Society 5.0 initiative aims to improve population well-being by fostering healthier dietary habits [1]. However, unhealthy eating behaviors remain widespread among young adults, including solitary eating, limited awareness of nutrition–health relationships, and extreme dieting practices. This emerging polarization between balanced and unbalanced eaters refers to a widening divergence in habitual dietary patterns, where some individuals maintain relatively nutrient-rich, balanced diets while others exhibit comparatively unbalanced or poor dietary behaviors. For example, trends in food group intake among young Japanese women have shown decreases in fruit and dairy consumption and increases in intake of meat and soft drinks across demographic subgroups, indicating heterogeneous dietary behaviors within this population [2,3]. This polarization in eating patterns is concerning, as eating patterns formed during youth have long-term implications for health and may contribute to future healthcare burdens [3,4]. Dietary behavior is shaped by complex and often unconscious processes, and positive changes are difficult to sustain [5,6]. Traditional Japanese health education—centered on knowledge delivery and compliance—has been criticized for its limited effectiveness in promoting lasting behavioral change [7]. Consequently, interest has grown in behavioral science-based strategies that encourage voluntary and sustainable improvements in dietary behavior [8].

Recent research has explored nudging, defined as subtle modifications to the choice environment that promote healthier decisions without restricting autonomy [9], as a strategy for improving dietary behavior [10,11,12,13,14,15,16,17]. In this study, a nudge refers to an environment structured to facilitate healthier dietary choices, regardless of whether users consciously recognize the guidance. Although nudges can be effective, meta-analytic evidence indicates that single nudges typically have modest impacts, whereas multifaceted strategies yield more robust behavioral change [18].

To strengthen nudging approaches, this study incorporates gamification, defined as the application of game mechanics to non-game contexts to enhance engagement, motivation, and behavioral change [19]. While diet management apps such as Asken, CALOMEAL, FiNC, OWN, and YAZIO [20,21,22,23,24] employ gamified features, they predominantly attract individuals who are already motivated or are engaged in medically supervised dieting. Users with low intrinsic motivation or limited dietary skills tend to disengage early. Gamification may help engage such populations. Stehr et al. identified MyFitnessPal user subgroups—including “indifferent individuals” and “socializers”—illustrating how entertainment and social interaction can sustain usage [25]. Empirical studies also indicate that gamified dietary interventions are most effective when integrating elements such as achievement, competition, social interaction, and enjoyment [26,27,28,29,30]. Incorporating these elements is considered essential for promoting engagement and reducing dropout in dietary management interventions [31].

This study utilizes FoodLog Athl [32], a nutrition management application developed by the Aizawa and Yamakata Laboratory at the University of Tokyo to support communication between athletes and dietitians [33]. To examine gamification as a nudging strategy, two feedback features were added based on the Dietary Reference Intakes for Japanese [34]: (1) a star-based rating system representing adherence to personalized nutrient targets, and (2) a radar chart visualizing energy and nutrient balance. These features operationalize the gamification principle of achievement and may be particularly effective for young adults, who often have limited exposure to dietary counseling and low engagement with traditional health information.

Theoretical frameworks for health behavior change provide tools for designing targeted interventions and clarifying structural determinants of behavior. The present study employs the Theory of Planned Behavior (TPB) [35], an extension of the Theory of Reasoned Action [36], which has been widely applied to dietary behavior. Prior research indicates that perceived behavioral control is particularly influential for unhealthy eating and that age moderates intention–behavior associations [37], underscoring the importance of contextualized applications of TPB. In recent theoretical developments, the Theory of Reasoned Goal Pursuit (TRGP) has been proposed as an extension of the Theory of Planned Behavior, emphasizing that attitudes and subjective norms are motivationally relevant primarily when they are aligned with currently active goals [38]. While the present study is grounded in the TPB framework, this goal-oriented perspective provides a useful conceptual background for interpreting belief- and norm-related processes underlying dietary behavior. In this study, the Theory of Planned Behavior (TPB) was used as the core conceptual framework to explain healthy dietary choice behavior. TPB constructs—attitude toward the behavior, subjective norm, self-efficacy (conceptualized as perceived behavioral control), intention, and behavior—were operationalized as measurable variables using a structured questionnaire and examined in relation to dietary behavior using composite-score-based path analysis.

This study is innovative in that it applies a composite-score-based path analysis within an SEM framework to clarify how core TPB components relate to everyday healthy dietary choice behavior in young Japanese adults, while maintaining a parsimonious, theory-driven specification. In addition, by treating star-rating feedback as a contextual nudging feature rather than an independent TPB determinant, the study offers exploratory insights into how gamified evaluative cues may shape reflective awareness and self-efficacy within the same theoretical structure.

Therefore, this study aimed to (1) develop and test a TPB-based model of healthy dietary choice behavior among young Japanese adults, including moderator effects, and (2) evaluate the influence of gamification—implemented through a star-rating nudge in FoodLog Athl—on dietary choice behavior and its role within the model. In line with national guidelines [4], a healthy diet in this study is conceptualized as nutritionally balanced, providing adequate energy and protein intake and including diverse foods such as seafood, legumes, dairy products, vegetables, and fruits, regardless of dining context (home-cooked meals, eating out, or delivered foods).

## 2. Materials and Methods

### 2.1. Sample

Between 2 and 9 October 2024, 188 participants aged 18–39 were recruited through snowball sampling via consortium networks and university-affiliated contacts, and completed an online pre-survey using Google Forms after providing written informed consent.

Prior to participation, participants were required to read a detailed written explanation of the study provided as a PDF document, and consent was obtained electronically by actively checking an “I agree” box; participants who did not provide consent were unable to proceed to the questionnaire.

Questionnaire data were collected using Google Forms as a temporary data collection tool, with access restricted to the research team via password-protected institutional Google accounts. After data collection was completed, the data were promptly downloaded, stored on secure, access-controlled university servers, and subsequently deleted from the Google Forms platform.

No incentives were provided. All responses were complete, with no missing data. Participants were distributed across several prefectures, with higher concentrations in Shiga (*n* = 85), Osaka (*n* = 32), Kyoto (*n* = 28), Tokyo (*n* = 12), Aichi (*n* = 8), Miyagi (*n* = 5), and Hyogo (*n* = 5), reflecting the geographic characteristics of participating universities. This study was conducted in accordance with the Declaration of Helsinki and approved by the Ethical Review Board of Ritsumeikan University (Approval No. BKC-LSMH-2024-018).

### 2.2. Survey Questionnaire Development

Survey items were developed based on prior studies on dietary choices, mobile meal planning, and gamification [3,6,26,39] and incorporated constructs from the Theory of Planned Behavior (TPB). Normative factors reflecting beliefs about appropriate dietary decision-making were included to examine their influence on the attitude–behavior process [40].

A preliminary survey (*n* = 220; ages 18–70) conducted between 23 July and 2 August 2024 informed scale refinement. The preliminary survey included a larger number of participants and a broader age range, as it was conducted as an exploratory phase for questionnaire refinement and model development. Exploratory factor analysis (maximum likelihood, Promax rotation) was conducted on nine subscales. Items with factor loadings <0.40 or insufficient observed variables were removed. The final questionnaire contained 16 TPB items and 10 normative items (Table 1).

#### 2.2.1. Subscales

- Cognitive (CO)

Six items were selected from prior studies [3,6,26,39] and adapted from food literacy topics designated as essential by the Ministry of Agriculture, Forestry and Fisheries. These items were adapted from food literacy topics designated as essential by the Ministry of Agriculture, Forestry and Fisheries. Because many of these topics are not widely known among young adults, responses were heterogeneous, resulting in low internal consistency (α = 0.38 in the main study; *α* = 0.53 in the preliminary survey). Given their educational and exploratory nature, cognitive items were retained for descriptive reporting but excluded from moderation analyses and were not incorporated as core constructs in path or conditional process models, as their limited reliability may attenuate inferential validity. Responses were coded as True/False/Do not know.

- Attitude toward the Behavior (ATB)

ATB assessed evaluative orientations toward balanced dietary choices according to Ajzen’s official guidance [41] using five items adapted from the stages of behavior change (precontemplation, contemplation, preparation, action, and maintenance) [42,43]. Responses were rated on five-point bipolar adjective scales. One item (ATB3) demonstrated limited psychometric contribution during preliminary analysis and was removed. The resulting two-item scale exhibited low internal consistency (*α* = 0.51), although retained items adequately captured the attitudinal content domain, and this scale was therefore interpreted with caution in inferential analyses.

- Subjective Norm (SN)

SN assessed perceived social pressure on dietary behavior using three items rated on five-point bipolar scales. Reliability was acceptable (*α* = 0.70).

- Self-Efficacy (SE)

SE measured confidence in maintaining a balanced diet [44]. Although perceived behavioral control (PBC) and SE were initially assessed separately, factor analysis merged PBC items with behavior and ascription of responsibility. Given evidence that SE is a stronger predictor of health behaviors than PBC [45,46], SE was adopted. Four items on five-point scales demonstrated acceptable reliability (*α* = 0.72), and SE was therefore interpreted as a motivational belief reflecting perceived capability rather than situational control constraints.

- Intention (INT)

INT assessed readiness to engage in healthy dietary behavior through three statements rated on five-point scales. Reliability was acceptable (*α* = 0.70).

- Behavior (BE)

BE assessed actual healthy dietary choice behavior using four statements rated on five-point scales, including two items reclassified from PBC. Reliability was acceptable (*α* = 0.72).

- Awareness of Consequences (AC)

AC assessed perceived negative outcomes of poor dietary habits [47] using three items rated on five-point scales. Reliability was acceptable (*α* = 0.75).

- Ascription of Responsibility (AR)

AR measured perceived personal responsibility for dietary decisions [48] using three items, including one reassigned from PBC. Reliability was marginal but acceptable (*α* = 0.66).

- Personal Norm (PN)

PN assessed personal obligations based on internal standards [49] using four items rated on five-point scales. Reliability was acceptable (α = 0.70).

#### 2.2.2. Demographic Variables

Participants indicated whether they considered themselves the primary “person making dietary choices (PDC)” using a five-point scale. Additional demographic variables included gender, age, occupation, university year/graduate status, marital status, living situation, economic sufficiency, frequency of smartphone game use, meal-management app use, and weekly meal frequency. Variables 1–9 were evaluated as potential moderators. Except for demographic and cognitive items, all items were randomized to minimize response bias.

### 2.3. Distribution of FoodLog Athl and Participant Experience

**Figure 1:**
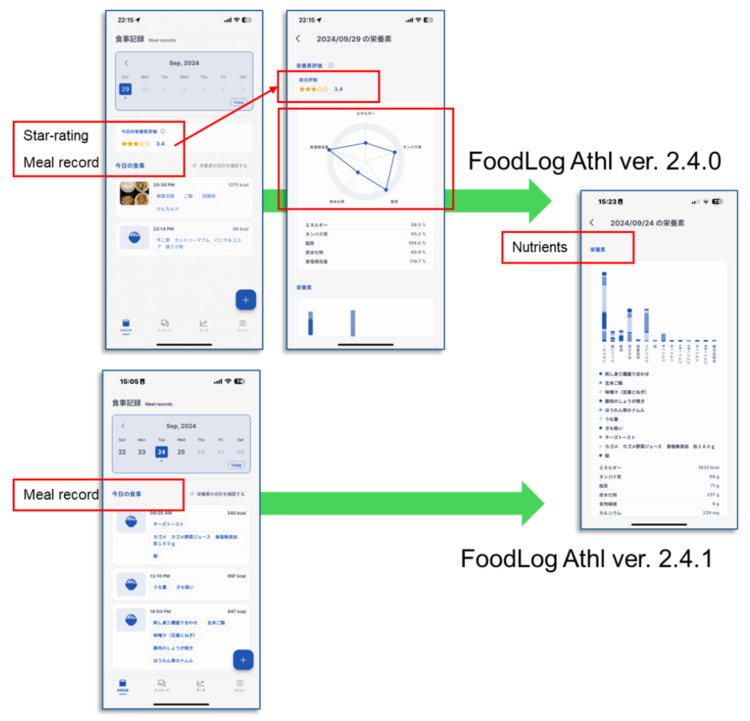
Interfaces of FoodLog Athl version 2.4.0 (star-rating feature) and version 2.4.1 (nutritional-value display only). Japanese labels indicate meal record, daily meal list, nutrient balance chart, and dietary quality star rating.

Participants were assigned—balancing gender, age, and living situation—to either an intervention or control group. The intervention group used FoodLog Athl v2.4.0, which included star-rating and radar-chart feedback, while the control group used v2.4.1, which displayed nutritional values only. FoodLog Athl is a mobile nutrition management application that allows users to record meals and review estimated energy and nutrient intake based on logged food items. Both groups used the app for seven days (Figure 1).

FoodLog Athl estimates nutritional content using a multimedia recipe dataset linked to the Standard Tables of Food Composition in Japan 2020 (Eighth Revised Edition) [50] and a nutrition estimation model (Appendix A.1). Following Nakamoto et al. [33], participants:

1. Installed the app via the Google Play or Apple Store.
2. Entered personal profile information (sex, birthdate, sport participation, and physical activity level) to generate individualized nutrient targets based on Dietary Reference Intakes for Japanese 2020 [34].
3. Logged all meals, snacks, and beverages for seven days.
4. Adjusted ingredient quantities following automated detection or manual search.
5. Reviewed intake graphs and adjusted entries to approach target values.

### 2.4. Statistical Analysis

#### 2.4.1. Assessment of Dietary Choice Behavior Components

Item responses were numerically coded: cognitive items (1 = correct, 0 = incorrect or unknown) and five-point scales (1 = least favorable to 5 = most favorable). Component scores were normalized as percentages of the maximum possible scores. Overall component scores were computed by combining scale items [51].

Group differences were examined using the Mann–Whitney *U* and Kruskal–Wallis tests. Spearman’s rank correlation (*ρ*) was used to assess associations.

#### 2.4.2. Conceptual Model Analysis

The conceptual model was examined using composite-score-based path analysis conducted within a structural equation modeling framework with maximum likelihood estimation. Although the constructs in the model represent theoretical latent concepts, the analysis was performed using composite (scale score) variables derived from validated item sets rather than latent variables explicitly modeled with measurement error.

Model fit was evaluated using multiple indices, including the chi-square statistic (χ^2^) and degrees of freedom, the Comparative Fit Index (CFI), the Tucker–Lewis Index (TLI), the Standardized Root Mean Square Residual (SRMR), and the Root Mean Square Error of Approximation (RMSEA) with its 90% confidence interval. Conventional cutoff criteria were applied to assess model adequacy (CFI and TLI ≥ 0.95, SRMR ≤ 0.08, RMSEA ≤ 0.06) [52,53].

#### 2.4.3. Conditional Process Analysis

Moderation analyses examined whether demographic characteristics and normative factors (AC, AR, PN) moderated predictor–outcome relationships [54]. The pre- and post-survey models were estimated separately using regression-based path analysis within a conditional process framework that does not require sample stratification, rather than a multi-group SEM, because the aim was to examine how contextual conditions influence specific paths within the same theoretical model, not to compare different groups or test structural invariance. Conditional direct and indirect effects were estimated using PROCESS v4.2 for SPSS macro accessed via Hayes’s official distribution [55], with mean-centered variables. Statistical significance was set at *p* < 0.05. Analyses were performed using IBM SPSS Statistics 29, and path diagrams were generated using AMOS 29.

## 3. Results

### 3.1. Sample Characteristics

Participant characteristics are summarized in Table 2. The sample was predominantly male (73%), with 25% female and 1.1% non-responses. More than 80% were aged 18–29 years, and approximately 60% were second-year university students. Most participants were unmarried (90%) and 53% lived alone; 38% perceived their economic situation as insufficient. Nearly 80% reported using or owning mobile game applications, whereas only 14% used or owned diet-management apps.

### 3.2. Overall Assessment of Dietary Choice Behavior Components

Significant differences in dietary choice behavior components and normative factors were observed across stages of attitude toward the behavior (ATB), as well as according to age and living situation. Detailed mean comparisons and subgroup differences are provided in Supplementary Table S1.

### 3.3. Assessment of FoodLog Athl Experiment

#### 3.3.1. Changes in Sample Size

Of the 183 eligible participants (excluding those with invalid email addresses), approximately 30% downloaded the app, 60–70% logged meals, and about 20% completed the post-survey (see Supplementary Table S2). Approximately 70–80% of post-survey responses were successfully matched with dietary records. Thus, the final analytic sample consisted of 41 participants who completed the post-survey, including 25 users of the star-rating app version and 16 users of the non-rating version.

Participant records were matched across the pre-survey, app download, and post-survey stages using registered email addresses. When automatic matching was incomplete, participants were contacted individually to confirm record linkage. For participants whose records could be successfully matched, exploratory comparisons of basic demographic characteristics (age, sex, and living situation) did not indicate marked differences between those who completed the post-survey and those who were lost to follow-up. However, because such information was not available for all discontinued cases, formal statistical attrition analyses were not conducted.

#### 3.3.2. Mean Comparisons: Pre/Post Surveys and App Version

Mean comparisons revealed several significant differences between app versions (Table 3). Self-efficacy (SE) was significantly lower among star-rating users than non-rating users (45% vs. 57%, *p* = 0.010). For behavior (BE), pre-survey scores were significantly higher than post-survey scores (55% vs. 48%, *p* = 0.027), and non-rating users scored higher than star-rating users in the post-survey (55% vs. 44%, *p* = 0.029). Awareness of consequences (AC) was marginally higher among star-rating users than non-rating users, although this difference did not reach statistical significance (91% vs. 79%, *p* = 0.059). No other components or normative factors differed significantly.

#### 3.3.3. Assessment of Nutrient Rating Score

A total of 41 participants logged meals during the study period (star-rating: *n* = 21; non-rating: *n* = 20). As shown in Table 4, the number of logging days did not differ significantly between the star-rating and non-rating groups (M = 7.2 vs. 6.2 days, *p* = 0.307). In contrast, the star-rating group showed significantly higher overall nutrient rating scores than the non-rating group (M = 2.37 vs. 2.07, *p* = 0.032). In addition, the star-rating group scored significantly higher on all nutrient-specific ratings, including energy, protein, fat, carbohydrate, and salt (all *p* ≤ 0.009).

### 3.4. Assessment of the Dietary Choice Behavior Model

#### 3.4.1. Intercorrelations

Table 5 presents the intercorrelations among the model components. Attitude toward the behavior (ATB) was positively correlated with subjective norms (SNs) and intention (INT) (*r* = 0.34 and 0.52, respectively; *p* < 0.001), as well as with all normative factors, including awareness of consequences (AC), ascription of responsibility (AR), and personal norms (PNs). SNs showed moderate correlations with self-efficacy (SE), INT, behavior (BE), and all normative factors (*r* = 0.25–0.56; all *p* < 0.001). SE was significantly correlated with INT, BE, and PNs (*r* = 0.30–0.65; all *p* < 0.001). INT was moderately correlated with BE (*r* = 0.32, *p* < 0.001) and with all normative factors. In addition, the normative factors (AC, AR, and PN) were significantly intercorrelated.

#### 3.4.2. Path Analysis (SEM Framework)

**Figure 2:**
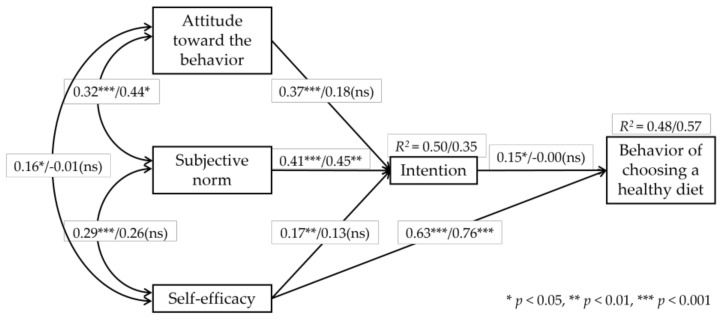
Standardized regression estimates of the dietary choice behavior model in the pre-survey (*n* = 188) and post-survey (*n* = 41).

Path analysis results, estimated within a structural equation modeling framework, are presented in Figure 2 and Table 6. Given the relatively small sample size for the post-survey path analysis (*n* = 41), the model was specified parsimoniously, consistent with previous methodological recommendations [56,57,58]. In the pre-survey model (*n* = 188), intention and self-efficacy were significant predictors of dietary choice behavior, with intention (*β* = 0.15, *p* = 0.010) and self-efficacy (*β* = 0.63, *p* < 0.001) jointly explaining 48% of the variance in behavior. Intention was significantly predicted by attitude toward the behavior (ATB; *β* = 0.37, *p* < 0.001), subjective norm (SN; *β* = 0.41, *p* < 0.001), and self-efficacy (SE; *β* = 0.17, *p* = 0.002), accounting for 50% of the variance in intention, with SN emerging as the strongest predictor.

In the post-survey model (*n* = 41), self-efficacy remained a strong and significant predictor of dietary choice behavior (*β* = 0.76, *p* < 0.001), explaining 57% of the variance in behavior. In contrast, intention was no longer significantly associated with behavior (*β* = −0.00, *p* = 0.968). Intention was significantly predicted by subjective norm (SN; *β* = 0.45, *p* = 0.003), whereas the effects of attitude toward the behavior (ATB; *β* = 0.18, *p* = 0.215) and self-efficacy (SE; *β* = 0.13, *p* = 0.339) were not statistically significant. Overall, the post-survey model accounted for 35% of the variance in intention.

Both the pre- and post-survey models showed good overall fit, as indicated by the chi-square statistic (χ^2^ (*df* = 2) = 0.36 and 0.42, respectively), TLI ≥ 1.03, NFI ≥ 0.99, CFI = 1.00, SRMR ≤ 0.02, and RMSEA = 0.00 with acceptable 90% confidence intervals, as shown in Table 6.

#### 3.4.3. Moderation Analysis

Moderation analyses based on mean-centered variables are summarized in Table 7 (*n* = 188). A significant interaction between gender and subjective norms (SNs) was observed in predicting intention, indicating that the association between SNs and intention differed by gender (*b*_3_ = 0.340, 95% CI = 0.084–0.596, *p* = 0.009). Simple slope analyses showed a stronger SN–intention association among males (*b* = 0.589) than females (*b* = 0.249).

In addition, moderated mediation analyses revealed that the indirect effect of self-efficacy on behavior via intention varied as a function of several moderators. The intention–behavior association was significantly moderated by university year (*b*_2_ = −0.168, 95% CI = −0.323 to −0.013, *p* = 0.034) and by diet app use frequency (*b*_2_ = −0.211, 95% CI = −0.394 to −0.028, *p* = 0.024). Furthermore, awareness of consequences (AC) and ascription of responsibility (AR) significantly moderated the path from self-efficacy to intention, with higher levels of AC (*a*_3_ = 0.009, *p* = 0.024) and AR (*a*_3_ = 0.015, *p* < 0.001) strengthening this association. Conditional indirect effects were significant only at higher levels of AC and AR.

## 4. Discussion

This study developed and tested a conceptual model of healthy dietary choice behavior among young Japanese adults by integrating the Theory of Planned Behavior (TPB) with self-efficacy and additional normative factors. The model accounted for 48% of the variance in dietary behavior and 50% of the variance in intention, consistent with effect sizes reported in previous TPB research [35]. These findings support the theoretical coherence of the model and its applicability to dietary behavior in this population.

From a theoretical perspective, the present findings suggest that gamified feedback does not operate as an independent determinant within the Theory of Planned Behavior, but rather as a contextual mechanism that shapes how core TPB constructs are experienced and translated into action. Specifically, star-rating feedback appears to influence reflective awareness and evaluative standards, which may temporarily recalibrate self-efficacy and perceived behavioral difficulty without directly altering underlying attitudes or intentions. In this sense, gamification can be understood as a situational modifier that affects the salience and interpretation of beliefs, rather than as a distinct motivational construct.

Similarly, the observed moderation effects of gender, university year, and living situation are not conceptualized as structural differences in the TPB model itself, but as contextual conditions that influence the strength with which intentions are enacted. These factors likely shape daily decision environments—such as autonomy over meals, exposure to normative cues, and reliance on external guidance—thereby modulating intention–behavior consistency without necessitating separate latent structures or multi-group model comparisons.

### 4.1. Predictors of Intention: The Prominence of Subjective Norms

Subjective norms emerged as the strongest predictor of intention, surpassing both attitude toward the behavior and self-efficacy. This contrasts with meta-analytic evidence indicating that attitude typically represents the dominant predictor of intention [39,59,60]. A gender moderation effect further revealed that the subjective norm–intention association was significantly stronger among males. Prior research shows that social expectations exert particularly strong influences on male adolescents and young adults [61], suggesting that normative pressures may be especially relevant when daily meals are often taken alone—as was the case for many participants.

Interpretation of the attitude–intention link warrants caution due to limited internal consistency in the ATB scale (Cronbach’s *α* = 0.51). Item count was constrained by field implementation requirements, and although the retained ATB items preserved content validity, reduced reliability likely attenuated the observed effect size, and the corresponding path estimates should therefore be interpreted as conservative. This may partially explain the comparatively smaller contribution of attitude within the model.

From a broader communication perspective, the present findings may also be interpreted in light of research on attribute framing in food choice. A recent meta-analysis by Dolgopolova et al. (2022) demonstrated that gain-framed food attributes generally exert stronger effects on consumers’ attitudes than loss-framed attributes, whereas effects on behavioral intentions tend to be weaker and more context-dependent [62]. Although the internal consistency of the attitude toward the behavior (ATB) scale was limited in the present study, the comparatively weaker role of attitude is consistent with this broader literature, which suggests that attitudinal responses are particularly sensitive to how health-related information is framed. Although attribute framing was not experimentally manipulated in the current study, these findings highlight the potential importance of communication strategies that emphasize positive, goal-relevant outcomes for shaping attitudinal and motivational precursors of healthy dietary behavior.

### 4.2. Role of Self-Efficacy in Predicting Behavior

Self-efficacy consistently emerged as the strongest predictor of dietary behavior across all datasets (pre-, post-, and preliminary surveys). This aligns with Bandura’s theoretical work demonstrating that self-efficacy facilitates behavioral persistence, initiation, and resilience [44]. Furthermore, the effect of self-efficacy on intention was amplified under higher awareness of consequences and ascription of responsibility, indicating that motivational cognitions are strengthened when individuals perceive both personal risk and personal responsibility. Similar patterns have been observed in digital and gamified dietary interventions, where self-efficacy often functions as a core mechanism of behavioral engagement [26,39]. In this study, participants with the most positive dietary attitudes also had the highest self-efficacy scores, underscoring the importance of interventions that foster confidence in managing dietary choices. In this context, from the perspective of the Theory of Reasoned Goal Pursuit, attitudes and normative influences are expected to shape motivation and intention primarily when relevant goals are activated. Although goal activation was not directly measured in the present study, the normative and belief-related variables included in our model—such as awareness of consequences, ascription of responsibility, and personal norms—may be interpreted as goal-relevant beliefs that become motivationally salient when health-related goals are chronically or situationally activated. In this sense, TRGP offers a complementary interpretive framework that helps contextualize our findings without extending beyond the scope of the current design.

### 4.3. Moderation of the Intention–Behavior Relationship

The intention–behavior relationship was moderated by university year and diet app usage. Lower-year students demonstrated stronger intention–behavior consistency, whereas higher-year students showed weaker effects. This may reflect life-stage differences, with early-year students having fewer competing obligations and greater openness to dietary guidance. Participants who rarely used diet apps also showed stronger intention–behavior translation than frequent users, suggesting that individuals with less prior exposure to dietary management may rely more directly on intention when making food choices (e.g., selecting cafeteria meals or convenience-store options).

Given that 81% of respondents lived alone, 86% did not use diet apps, and 73% made autonomous dietary decisions, these contextual factors likely shaped how intentions translated into actions.

### 4.4. Effects of the Gamified FoodLog Athl Intervention

It should be noted that the present analyses were not designed to statistically test intervention effects or pre–post differences in path coefficients. Given the limited post-intervention sample size, the following findings should be interpreted as exploratory patterns rather than confirmatory evidence. Compared with the non-rating version, the star-rating group was observed to show:

- Lower self-efficacy and behavior scores, suggesting that objective feedback may initially highlight the difficulty of achieving nutritional balance and temporarily reduce perceived competence;
- Slightly higher (near-significant) awareness of consequences (*p* = 0.059), indicating elevated recognition of dietary risks.

These trends parallel findings from prior dietary self-monitoring studies, in which external feedback increases awareness but may temporarily lower self-evaluation as individuals calibrate their expectations [6], particularly when evaluative standards are externally defined rather than internally generated. Qualitative comments confirmed that users became particularly aware of dietary imbalances when confronted with low scores or missing nutrients. Consistent with these perceptions, participants in the star-rating group also demonstrated more favorable nutrient rating scores, suggesting that heightened awareness may translate into more nutritionally balanced food choices even when self-evaluations are temporarily lowered.

While the moderation effect of the star-rating feature on model pathways was not statistically significant—likely due to the limited post-intervention sample size—supplementary conditional process analyses showed that the magnitude of the direct effect of self-efficacy on behavior was descriptively larger in the star-rating group than in the non-rating group. Specifically, the standardized coefficient was higher in the star-rating group (*β* = 0.762, *p* < 0.001) than in the non-rating group (*β* = 0.641, *p* = 0.003). This pattern may indicate that if users develop or regain confidence, gamified evaluative feedback may facilitate the translation of self-efficacy into action.

### 4.5. Qualitative Experiences of Gamified Feedback

Open-ended responses further contextualized these quantitative findings. Most star-rating users (76%, *n* = 19 of 25) evaluated the feature positively, describing heightened awareness of dietary balance when receiving low ratings or visualizations of nutrient deficits. Reflection was frequently triggered by snacking, unexpectedly low scores, or recognition of missing nutrients.

Patterns of app engagement differed by group. Non-rating users tended to rely on bodily sensations or perceived physical changes, whereas star-rating users emphasized accurate meal logging and receiving personalized guidance. These distinctions suggest that gamified feedback may cultivate a more analytical and self-reflective mindset toward dietary behavior, particularly among individuals with limited prior knowledge or lower self-efficacy. Although interaction effects were statistically nonsignificant, the pattern of stronger direct self-efficacy effects among star-rating users supports this interpretation.

### 4.6. Broader Context: Solitary Eating and Young Adults’ Dietary Health

This study also highlights broader contextual challenges. Solitary eating and breakfast skipping were common, consistent with national trends indicating that single-person households are increasing and projected to reach 44% by 2050 in Japan [63]. Such demographic changes reduce opportunities for shared meals, which traditionally provide social and normative cues that scaffold healthy eating. In this study, 78% ate breakfast alone, and more than one-third ate lunch or dinner alone; additionally, 32% regularly skipped breakfast (Table A1). Solitary eating is associated with lower diet quality, weaker mealtime routines, and elevated risks such as frailty [64]. In such environments, gamified diet-management applications may serve as compensatory mechanisms that provide feedback, structure, and subtle normative cues absent from users’ daily routines.

### 4.7. Implications for Gamification-Based Dietary Interventions

For individuals who do not naturally prioritize dietary management, gamification may offer a sustainable engagement route. Nutrient scoring, ranking systems, avatar or pet-based progression, and social interaction features can attract users who initially engage for entertainment rather than health improvement [25]. Over time, repeated exposure to nutritional feedback may support healthier decision-making through incremental learning about dietary balance. If apps successfully enhance users’ understanding of energy and nutrient sufficiency, they may ultimately promote autonomous dietary planning beyond the app environment.

## 5. Conclusions

This study developed and evaluated an extended Theory of Planned Behavior (TPB)–based model of healthy dietary choice behavior among young Japanese adults, using composite-score-based path analysis and incorporating self-efficacy and additional normative constructs. The model demonstrated substantial explanatory capacity, with self-efficacy emerging as the strongest predictor of behavior and, together with intention, accounting for nearly half of the variance in healthy dietary choices. Moderation analyses highlighted the importance of contextual factors: subjective norm more strongly predicted intention among males; the intention–behavior association differed by university year and diet app use; and the influence of self-efficacy on intention was amplified when awareness of consequences and ascription of responsibility were higher. These findings underscore the central role of self-efficacy and the importance of personal and situational contexts in shaping dietary decision-making.

The seven-day gamified intervention using FoodLog Athl provided further insights. Although moderation effects of the star-rating feature were not statistically significant—likely in part due to the limited post-intervention sample—users who received star-based feedback demonstrated more favorable nutrient balance and heightened awareness of dietary risks. At the same time, they reported lower self-efficacy and behavior scores, suggesting that objective feedback may initially prompt critical self-reflection before supporting longer-term confidence building. The larger conditional direct effect of self-efficacy on behavior observed among users exposed to star-rating feedback indicates the potential of gamified evaluative feedback to strengthen behavioral engagement once users adapt to the feedback system.

Overall, this study contributes empirical evidence for the integration of behavioral theory and gamification in promoting healthier dietary choices among young adults. The findings highlight the need to enhance self-efficacy, address normative influences, and tailor interventions to user characteristics and lifestyle contexts. Future research with larger and more diverse samples, improved attitudinal measurement, and longer intervention durations is warranted to refine the model and more robustly assess the effectiveness of gamified dietary feedback systems.

### Limitations and Future Directions

This study has several limitations that warrant consideration. First, the use of snowball sampling resulted in a sample that was geographically concentrated and predominantly male, limiting the generalizability of the findings. Because recruitment relied primarily on university consortium networks, the sample may overrepresent students in STEM-oriented environments, which likely contributed to the male-skewed composition. Although the present results provide valuable insights into dietary decision-making among young adults who often live alone and exercise substantial autonomy over their meals, future studies should recruit more diverse samples across regions, socioeconomic backgrounds, and age groups to enhance external validity.

Second, the internal consistency of the attitude toward the behavior (ATB) scale was relatively low (Cronbach’s *α* = 0.51). Practical constraints during field deployment restricted the number of items that could be included, and one item was removed based on preliminary psychometric analysis and established guidance for TPB measurement. Although content validity for the attitudinal domain was preserved, the reduced reliability necessitates cautious interpretation of ATB-related pathways within the structural equation model, particularly given that composite scores rather than latent variables were used. Future research should expand the ATB item pool and conduct more rigorous validation to improve measurement precision.

Third, the post-intervention sample size was small due to technical issues with account linking and varying levels of engagement with the FoodLog Athl app. Consequently, moderation effects and pre–post comparisons should be regarded as exploratory. The limited sample also increases the risk of unstable parameter estimates and may inflate model-fit indices in models with few degrees of freedom. To address this, future interventions should employ improved participant tracking, enhanced platform stability, and recruitment strategies designed to increase retention.

Fourth, the intervention period was restricted to seven days, likely capturing only early-stage responses to the gamified feedback rather than sustained behavioral change. Short-term exposure may heighten awareness while temporarily lowering perceived competence, as observed in the star-rating group. Longer-term, multi-phase interventions are needed to determine whether early declines in self-efficacy give way to improved confidence and more stable dietary behaviors as users adapt to the feedback system.

Taken together, future work should prioritize:

(a) Developing more psychometrically robust TPB and normative measures, including expanded ATB items;
(b) Designing longer-term gamified interventions capable of maintaining engagement among users with low intrinsic dietary motivation;
(c) Incorporating objective digital indicators to monitor real-time behavioral adjustments.

Addressing these limitations will support a more comprehensive evaluation of how gamified feedback can promote sustained improvements in dietary behavior and refine theoretical models of young adults’ dietary decision-making.

## Figures and Tables

**Table 1:** Summary of mean, standard deviation, and Cronbach’s α for components of the dietary choice behavior model (*n* = 188).

Predictor (Abbreviation)	*α*	**Item No.** ^a^	Question Items ^a^
Cognitive ^b^ (CO)	0.38	CO1	Japan’s food self-sufficiency rate is 68%. (False)
		CO2	Japanese people eat more seafood than meat. (False)
		CO3	In recent years, the protein intake of Japanese people has exceeded the standard value. (False)
		CO4	Climate change is said to affect nutritional disparities. (True)
		CO5	Protein is contained not only in meat, fish, eggs, and soybean products, but also in many other foods, such as staple foods such as rice, bread, and noodles, and side dishes such as vegetables and mushrooms. (True)
		CO6	Calories in food, nutritional balance, and eating a variety of foods are related to a healthy lifespan. (True)
Attitude toward the behavior (ATB)	0.51	ATB1 ^c^	Which of the following describes your level of interest in your diet? 1. I’m not interested in my diet and have no intention of improving it. 2. I’m interested in my diet, but I think it’s fine the way it is even if it’s a little unbalanced. 3. I’m interested in my diet, so I’d like to improve it if the opportunity arises. 4. I’m interested in my diet and have been trying to improve it for less than 6 months. 5. I’m already practicing a better diet and continuing it.
		ATB2	I have little interest in my diet and have no intention of improving it. (Five-point scale)
Subjective norm (SN)	0.70	SN2	If someone I respect recommends eating a healthy diet, I think I will do so.
		SN3	If the people around me pay attention to a balanced diet, I think I should do the same.
		SN4	If someone important to me gives me advice, I will eat a healthy diet.
Self-efficacy (SE)	0.72	SE1	It is easy for me to set a goal to eat healthy food and achieve it.
		SE2	I am confident that I can continue to eat healthy food even when I am busy and short on time.
		SE3	I am confident that I can continue to eat healthy food on my days off.
		SE4	I don’t mind cooking my own meals.
Intention (INT)	0.70	INT1	I would like to learn more about healthy food if possible.
		INT2	I would try to eat healthy food if I knew how to do it.
		INT4	I try to eat healthy food for my health.
Behavior (BE)	0.72	BE1	It is difficult for me to always eat healthy food. (*R*) ^d^
		BE2	I always eat healthy food.
		BE3	When there is no healthy food nearby, I travel to a place where I can.
		BE4	How often do you forget to eat healthy food when you are busy? (*R*)
Awareness of consequences (AC)	0.75	AC1	If I neglect my diet daily, it will affect my future health.
		AC2	Eating habits affect my future health.
		AC3	I believe that if I neglect my diet because I am concerned about my appearance or style, it will affect my future health.
Ascription of responsibility (AR)	0.66	AR1	It’s up to me to eat healthy food.
		AR2	I am responsible for whether I eat healthy food or not.
		AR3	I am responsible for my own and my family’s healthy food.
Personal norm (PN)	0.70	PN1	I sometimes feel guilty when I don’t eat healthy food.
		PN2	I sometimes blame myself later for eating without considering nutritional balance.
		PN3	I think it is my duty to eat healthy food.
		PN4	I think I should eat healthy food even if it is a little troublesome.
Person who making dietary choices (PDC) ^e^	--	PDC	I can make my own dietary choices, and I make all the decisions about what meals I eat, including shopping, menus, and eating out. (Five-point scale)

^a^ To improve internal consistency, the following items were eliminated: ATB3 “I have already implemented and will continue better eating habits”; SN1 “The people important to me think I should eat a healthy diet”; INT3 “I will research new information about a healthy diet”; and BE5 “I can prepare healthy meals for myself.” ^b^ Cognitive was not a component of the dietary choice behavior model in this study, the internal consistency was not adjusted yet. ^c^ ATB1 is an item that examines the degree of indifference to interest in food choices using a five-stage model of behavioral change. ^d^ (*R*) indicates a reverse question. ^e^ Extract how many people are able to make their own decisions about dietary choices.

**Table 2:** Demographic characteristics of survey participants (*n* = 188).

Attributes		*n*	%
Gender	Male	138	73.4
	Female	48	25.5
	No answer	2	1.1
Age	18~29	157	83.5
	30s	31	16.5
Occupation	Office employee	26	13.8
	Student	153	81.4
	Educator and Researcher	5	2.7
	Self-employed	1	0.5
	Housewife	3	1.6
Students’ university year (*n* = 153)	1st	2	1.1
	2nd	109	58.0
	3rd	7	3.7
	4th	16	8.5
	Graduate school	19	10.1
	Others	35	18.6
Marital status	Married	14	7.4
	Unmarried	170	90.4
	Divorced	1	0.5
	N/A	3	1.6
Living situation	Living with spouse/partner	10	5.3
	Student Dormitory	4	2.1
	Living with family	72	38.3
	Living alone	100	53.2
	Living with someone except above (e.g., in a shared house)	2	1.1
Degree of economic sufficiency (5-point scale)	1 Not at all financially sufficiency	19	10.1
	2	52	27.7
	3	79	42.0
	4	31	16.5
	5 Very financially sufficiency	7	3.7
Usage of smartphone game applications	I always use it.	48	25.5
	I use it occasionally.	69	36.7
	I have game apps., but I rarely use them.	34	18.1
	I don’t use it.	29	15.4
	I have no plans to use it in the future.	8	4.3
Usage of smartphone diet management applications	I always use it.	7	3.7
	I use it occasionally.	7	3.7
	I have meal management apps., but I rarely use them.	12	6.4
	I don’t use it.	135	71.8
	I have no plans to use it in the future.	27	14.4

**Table 3:** Mean comparison of predictors and normative factors before and after the intervention and by app versions.

Surveys		ATB				SN				SE				INT			
	*n*	**M (%)**	SD	SE	*p*	M (%)	SD	SE	*p*	M (%)	SD	SE	*p*	M (%)	SD	SE	*p*
Pre-	188	66.2	15.9	1.2	0.096	71.3	16.6	1.2	0.958	54.1	17.5	1.3	0.097	76.1	14.8	1.1	0.850
Post-	41	70.2	12.1	1.9		72.5	16.4	2.6		49.6	16.3	2.5		75.6	12.3	1.9	
Post-survey, ratings:																	
Star-	25	70.4	11.7	2.3	0.556	73.1	17.3	3.5	0.087	44.8	15.8	3.2	0.010	76.0	12.3	2.5	0.817
Non-	16	70.0	13.2	3.3		71.7	15.5	3.9		57.2	14.4	3.6		75.0	12.5	3.1	
**Surveys**		**BE**				**AC**				**AR**				**PN**			
	***n***	**M (%)**	**SD**	**SE**	***p***	**M (%)**	**SD**	**SE**	***p***	**M (%)**	**SD**	**SE**	***p***	**M (%)**	**SD**	**SE**	***p***
Pre-	188	54.8	17.3	1.3	0.027	87.4	14.0	1.0	0.621	76.2	16.2	1.2	0.937	64.8	17.3	1.2	0.249
Post-	41	48.4	15.7	2.5		86.2	15.4	2.4		75.8	16.5	2.6		68.8	14.3	2.2	
Post-survey, ratings:																	
Star-	25	44.4	15.3	3.0	0.029	90.7	7.9	1.6	0.059	77.1	15.2	3.0	0.637	70.2	16.4	3.3	0.493
Non-	16	54.7	14.8	3.7		79.2	21.1	5.3		73.7	18.6	4.6		66.6	10.4	2.6	

**Table 4:** Mean comparison of nutrient rating scores between star-rating and non-rating groups (maximum score = 5 points).

Ratings		Recording Days for a Week					Overall Rating Max. 5 Points)				Energy				Protein			
*n* = 41	*n*	Days	M ^a^	SD	SE	*p*	M	SD	SE	*p*	M ^a^	SD	SE	*p*	M	SD	SE	*p*
Star-	21	151	7.2	3.12	0.68	0.307	2.37	1.10	0.09	0.032	0.59	0.29	0.02	0.001	0.91	0.51	0.04	<0.001
Non-	20	124	6.2	3.05	0.68		2.07	1.22	0.11		0.47	0.28	0.03		0.71	0.39	0.04	
**Ratings**			**Fat**				**Carbohydrate**				**Salt**							
*n* = 41	*n*		M	SD	SE	*p*	M	SD	SE	*p*	M	SD	SE	*p*				
Star-	21		0.85	0.54	0.04	0.005	0.51	0.26	0.02	0.009	0.77	0.52	0.04	0.001				
Non-	20		0.66	0.55	0.05		0.43	0.26	0.02		0.59	0.42	0.04					

^a^ Methodology for calculating star ratings in FoodLog Athl in Appendix A.1.

**Table 5:** Intercorrelations matrix of factors. (*n* = 188).

	M (%)	SD	ATB	*p*	**SN**	*p*	SE	*p*	INT	*p*	BE	*p*	AC	*p*	AR	*p*
Attitude toward the behavior	66.2	15.9	--													
Subjective norm	71.3	16.6	0.338	<0.001	--											
Self-efficacy	54.2	17.5	0.091	0.214	0.262	<0.001	--									
Intention	76.1	14.8	0.516	<0.001	0.561	<0.001	0.305	<0.001	--							
Behavior	54.8	17.3	0.077	0.292	0.250	<0.001	0.647	<0.001	0.320	<0.001	--					
Awareness of consequences	87.4	14.0	0.395	<0.001	0.432	<0.001	−0.008	0.912	0.487	<0.001	0.092	0.211	--			
Ascription of responsibility	76.2	16.2	0.298	<0.001	0.402	<0.001	0.115	0.117	0.323	<0.001	0.102	0.165	0.423	<0.001	--	
Personal norm	64.8	17.3	0.338	<0.001	0.470	<0.001	0.248	<0.001	0.470	<0.001	0.312	<0.001	0.270	<0.001	0.316	<0.001

Notes: -- indicates values not shown (upper triangle omitted).

**Table 6:** Standardized and unstandardized regression estimates for the dietary choice behavior model in the pre- and post-surveys, including model fit indices.

Constructs			Pre-Survey (*n* = 188)			Post-Survey (*n* = 41)		
			*β*	*b*	*p*	*β*	*b*	*p*
ATB	→	INT	0.37	0.35	<0.001	0.18	0.18	0.215
SN	→	INT	0.41	0.37	<0.001	0.45	0.34	0.003
SE	→	INT	0.17	0.14	0.002	0.13	0.10	0.339
SE	→	BE	0.63	0.62	<0.001	0.76	0.73	<0.001
INT	→	BE	0.15	0.17	0.010	−0.00	−0.01	0.968
Correlations			Corre.	Covar.		Corre.	Covar.	
ATB	–	SN	0.32	83.94	<0.001	0.44	86.38	0.010
ATB	–	SE	0.16	45.46	0.027	−0.01	−1.13	0.970
SN	–	SE	0.29	82.94	<0.001	0.26	67.20	0.114
			*R* ^2^			*R* ^2^		
		INT	0.50			0.35		
		BE	0.48			0.57		
Model fitness indices			Pre-survey			Post-survey		
		χ^2^ (*df*)	0.361(2)			0.419(2)		
		TLI	1.030			1.147		
		NFI	0.999			0.993		
		CFI	1.000			1.000		
		SRMR	0.007			0.017		
		RMSEA	0.000	90%CI = [0.000, 0.084] PCLOSE = [0.892]		0.000	90%CI = [0.000, 0.192] PCLOSE = [0.827]	

Notes: → indicates direction of regression path (predictor to outcome). *β* is standardized and *b* is unstandardized coefficient.

**Table 7:** Unstandardized OLS regression coefficients with confidence intervals estimating: (a) moderation of subjective norm by gender; and (b) conditional indirect effects of self-efficacy on behavior via intention moderated by university year, diet app use, awareness of consequences, and ascription of responsibility. All variables are mean-centered (*n* = 188).

SN-INT			Intention (*Y*)									
			Coeff.	SE	95% CI	*p*						
Subjective norm (*X*)	$b_{1}$	→	0.510	0.052	0.406, 0.613	<0.001						
Gender (*M*)	$b_{2}$	→	−2.662	1.762	−6.139, 0.815	0.133						
*X* × *M*	$b_{3}$	→	0.340	0.130	0.084, 0.596	0.009						
Constant	$i_{M}$	→	75.918	0.864	74.213, 77.622	<0.001						
			*R*^2^ = 0.371 *F*(3, 184) = 36.153, *p* < 0.001									
**SE-INT-BE**			**Intention (*M*)**						**Behavior (*Y*)**			
			**Coeff.**	**SE**	**95% CI**	***p***			**Coeff.**	**SE**	**95% CI**	***p***
Self-efficacy (*X*)	$a_{1}$	→	0.249	0.069	0.113, 0.385	<0.001	${c^{\prime }}_{1}$	→	0.640	0.063	0.516, 0.765	<0.001
Intention (*M*)							$b_{1}$	→	0.137	0.073	−0.006, 0.282	0.062
Students’ univ. year (*W*_1_) (*n* = 153)	$a_{2}$	→	0.309	1.056	−1.778, 2.396	0.770	${c^{\prime }}_{2}$	→	0.376	0.930	−1.461, 2.214	0.686
*X* × *W*_1_	$a_{3}$	→	−0.030	0.0716	−0.171, 0.111	0.675	${c^{\prime }}_{3}$	→	0.027	0.066	−0.103, 0.157	0.682
*M* × *W*_1_							$b_{2}$	→	−0.168	0.078	−0.323, −0.013	0.034
Constant	$i_{M}$	→	−0.054	1.161	−2.348, 2.239	0.963	$i_{Y}$	→	54.913	1.022	52.894, 56.933	<0.001
			*R*^2^ = 0.090 *F*(3, 149) = 4.914, *p* = 0.0028						*R*^2^ = 0.496 *F*(5, 147) = 28.953, *p* < 0.001			
Self-efficacy (*X*)	$a_{1}$	→	0.288	0.059	0.171, 0.404	<0.001	${c^{\prime }}_{1}$	→	0.607	0.057	0.496, 0.719	<0.001
Intention (*M*)							$b_{1}$	→	0.175	0.066	0.044, 0.306	0.009
Diet apps use (*W*_2_)	$a_{2}$	→	−0.635	1.461	−3.518, 2.249	0.665	${c^{\prime }}_{2}$	→	0.010	1.327	−2.608, 2.628	0.994
*X* × *W*_2_	$a_{3}$	→	−0.080	0.106	−0.290, 0.130	0.452	${c^{\prime }}_{3}$	→	0.003	0.096	−0.186, 0.193	0.972
*M* × *W*_2_							$b_{2}$	→	−0.211	0.093.	−0.394, −0.028	0.024
Constant	$i_{M}$	→	0.002	1.015	−2.000, 2.004	0.998	$i_{Y}$	→	54.768	0.914	52.965, 56.571	<0.001
			*R*^2^ = 0.126 *F*(3, 184) = 8.885, *p* < 0.001						*R*^2^ = 0.492 *F*(5, 182) = 35.241, *p* < 0.001			
Self-efficacy (*X*)	$a_{1}$	→	0.260	0.051	0.159, 0.361	<0.001	${c^{\prime }}_{1}$	→	0.616	0.061	0.495, 0.737	<0.001
Intention (*M*)							$b_{1}$	→	0.164	0.080	0.005, 0.323	0.043
Awareness of consequences (*W*_3_)	$a_{2}$	→	0.501	0.062	0.380, 0.623	<0.001	${c^{\prime }}_{2}$	→	−0.015	0.009	−0.190, 0.160	0.867
*X* × *W*_3_	$a_{3}$	→	0.009	0.004	0.001, 0.017	0.024	${c^{\prime }}_{3}$	→	0.003	0.004	−0.006, 0.002	0.552
*M* × *W*_3_							$b_{2}$	→	−0.001	0.005	−0.011, 0.008	0.764
Constant	$i_{M}$	→	0.009	0.849	−1.667, 1.684	0.992	$i_{Y}$	→	54.998	1.059	52.908, 57.088	<0.001
			*R*^2^ = 0.389 *F*(3, 184) = 38.980, *p* < 0.001						*R*^2^ = 0.478 *F*(5, 182) = 33.397, *p* < 0.001			
Self-efficacy (*X*)	$a_{1}$	→	0.203	0.054	0.095, 0.310	<0.001	${c^{\prime }}_{1}$	→	0.611	0.060	0.494, 0.729	<0.001
Intention (*M*)							$b_{1}$	→	0.144	0.075	−0.003, 0.292	0.055
Ascription of responsibility (*W*_4_)	$a_{2}$	→	0.299	0.058	0.184, 0.413	<0.001	${c^{\prime }}_{2}$	→	0.013	0.063	−0.111, 0.137	0.837
*X* × *W*_4_	$a_{3}$	→	0.015	0.003	0.008, 0.021	<0.001	${c^{\prime }}_{3}$	→	0.004	0.004	−0.003, 0.001	0.255
*M* × *W*_4_							$b_{2}$	→	0.000	0.004	−0.008, 0.008	0.975
Constant	$i_{M}$	→	−0.673	0.930	−2.501, 1.162	0.470	$i_{Y}$	→	54.642	0.975	52.719, 56.565	<0.001
			*R*^2^ = 0.285 *F*(3, 184) = 24.497, *p* < 0.001						*R*^2^ = 0.481 *F*(5, 182) = 33.783, *p* < 0.001			

Notes: → indicates regression equation direction (predictor to dependent variable; corresponds to the paths shown in Figure 2).

## Data Availability

The data presented in this study are available on reasonable request from the corresponding author. The data are not publicly available due to privacy and ethical restrictions associated with human subject information and app-linked dietary records.

## Supplementary Materials

The following supporting information can be downloaded at https://www.mdpi.com/article/10.3390/nu18040686/s1, Table S1: Comprehensive evaluation of components in the dietary choice behavior model; Table S2: Changes in sample size during the FoodLog Athl experiment (*n* = 183).

## Abbreviations

The following abbreviations are used in this manuscript:

PDC	Person making dietary choices
CO	Cognitive
ATB	Attitude toward the behavior
SN	Subjective norm
SE	Self-efficacy
INT	Intention
BE	Behavior
AC	Awareness of consequences
AR	Ascription of responsibility
PN	Personal norm

## Appendix A

### Appendix A.1. Methodology for Calculating Star Ratings in FoodLog Athl

Determination of target amounts for user profiles based on the “Dietary Reference Intakes for Japanese (2020)” [50].

(1) Calculated based on gender, age, and physical activity level entered in the user profile.
(2) The physical activity levels (PALs) are described as follows [34], p. 8:

- PALs of I, II, and III indicate low, medium, and high activity levels, respectively.
- PAL II indicates the physical activity level of people who can support themselves, and PAL I indicates the physical activity level of people who do not usually go outside. PAL I is the physical activity level that can be adapted to people who can support themselves in a nursing home.

#### Calculation of Fat and Carbohydrate Requirements

Fat and carbohydrate intake targets are calculated as percentages of an individual’s estimated energy requirement. The following formulas and assumptions are used:

Energy values:

- Fat 1 g = 9 kcal
- Carbohydrate 1 g = 4 kcal

Fat target

- The recommended fat intake is 20–30% of total energy intake (except for children under 1 year old).
- A standard value of 25% is typically used for adults.

Example:

For a 25-year-old male with an activity level of 2, the estimated energy requirement is 2650 kcal.

- Fat: 25% of 2650 kcal = 662.5 kcal
- Convert to grams: 662.5 kcal ÷ 9 kcal/g = 73.6 g of fat

Carbohydrate target

- The recommended carbohydrate intake is 50–65% of total energy intake (except for children under 1 year old).
- A standard value of 57.5% is typically used.

Example:

For the same individual (25-year-old male, activity level 2):

- Carbohydrates: 57.5% of 2650 kcal = 1523.75 kcal.
- Convert to grams: 1523.75 kcal ÷ 4 kcal/g = 380.9 g of carbohydrates.

Formula for calculating the overall rating (number of stars):

The scores for energy, protein, fat, carbohydrate, and salt equivalents are calculated using the following formulas (Equations (A1) and (A2)), and the average of these scores is used as the overall score.

(A1) $$Achievement\;rate:\;x=\frac{\left|Target\;value-Achivement\;value\right|}{Target\;value}$$

(A2) $$Score:s(x)=100\times exp\left(-{\left(\frac{x}{\alpha }\right)}^{2}\right)\;\;\;\;\;\alpha =0.5$$

## Appendix B

This appendix summarizes participants’ weekly meal frequencies and eating contexts (e.g., eating alone, with family, with peers), derived from the pre-survey dataset. The table provides descriptive information relevant to interpreting dietary behavior patterns.

**Table A1:** Weekly Frequency of Meal Intake and Dining Companions (*n* = 188).

Questions	Choices	Breakfast		Lunch		Dinner	
		*n*	%	*n*	%	*n*	%
Number of times per week you eat breakfast/lunch/dinner	Every day	74	39.4	145	77.1	161	85.6
	5–6 times a week	30	16.0	28	14.9	18	9.6
	3–4 times a week	24	12.8	10	5.3	7	3.7
	1–2 times a week	33	17.6	3	1.6	0	0.0
	Rarely	27	14.4	2	1.1	2	1.1
	Total	188	100	188	100	188	100
With whom you eat breakfast/lunch/dinner?	Rarely eat	10	5.3	2	1.1	9	4.8
	Alone	146	77.7	64	34.0	108	57.4
	Friend	1	0.5	101	53.7	1	0.5
	People at work	0	0.0	13	6.9	2	1.1
	Parent(s)	7	3.7	2	1.1	4	2.1
	Family	22	11.7	4	2.1	46	24.5
	Married couples	2	1.1	2	1.1	18	9.6
	Total	188	100	188	100	188	100

## References

[1] Cabinet Office, Secretariat of Science, Technology and Innovation Policy (2024). Cross-Ministerial Strategic Innovation Promotion Program (SIP): Building a Resilient and Nourishing Food Supply Chain Management for a Sustainable Future. https://www8.cao.go.jp/cstp/gaiyo/sip/sip_3/keikaku/01_foodchain.pdf.

[2] Matsumoto M., Tajima R., Fujiwara A., Yuan X., Okada E., Takimoto H. (2022). Trends in Food Group Intake According to Body Size among Young Japanese Women: The 2001–2019 National Health and Nutrition Survey. Nutrients.

[3] Ministry of Agriculture, Forestry and Fisheries (MAFF) (2019). Survey Results on Eating Habits of the Younger Generation. https://www.maff.go.jp/j/syokuiku/websurvey/attach/pdf/websurvey-2.pdf.

[4] Ministry of Education, Culture, Sports, Science and Technology (MEXT), Ministry of Health, Labour and Welfare (MHLW), Ministry of Agriculture, Forestry and Fisheries (MAFF) (2016). Interpretation Manual for the Dietary Guidelines. https://www.mhlw.go.jp/file/06-Seisakujouhou-10900000-Kenkoukyoku/0000132167.pdf.

[5] Hayashi F. (2023). Use of nudges to change dietary behavior: Usefulness and challenges in primary prevention, special article: Health education, health promotion and nudge theory: Trends and issues. Jpn. J. Health Educ. Promot..

[6] Watanabe-Ito M., Kishi E., Shimizu Y. (2020). Promoting healthy eating habits for college students through creating dietary diaries via a smartphone app and social media interaction: Online survey study. JMIR mHealth uHealth.

[7] Nakamura M. (2002). Health behavior change based on behavioral science: Theory and practice. Jpn. J. Nutr. Diet..

[8] Watanabe K., Kawakami N., Imamura K., Inoue A., Shimazu A., Yoshikawa T., Hiro H., Asai Y., Odagiri Y., Yoshikawa E. (2017). Pokémon GO and psychological distress, physical complaints, and work performance among adult workers: A retrospective cohort study. Sci. Rep..

[9] Thaler R.H., Sunstein C.R. (2008). Nudge: Improving Decisions about Health, Wealth, and Happiness.

[10] Buratto A., Lotti L. (2024). Encouraging sustainable food consumption through nudges: An experiment with menu labels. Ecol. Econ..

[11] Colleoni M., Rossetti M., Magatti G., Palestini P., Iannantuoni G. (2021). A nudging approach to promote healthier and more sustainable food consumption and lifestyles at the University of Milano-Bicocca. J. Sustain. Perspect..

[12] Kurz V. (2018). Nudging to reduce meat consumption: Immediate and persistent effects of an intervention at a university restaurant. J. Environ. Econ. Manag..

[13] Meeusen R.E., van der Voorn B., Berk K.A. (2023). Nudging strategies to improve food choices of healthcare workers in the workplace cafeteria: A pragmatic field study. Clin. Nutr. ESPEN.

[14] Murakami T., Hosoi N., Ohta M. (2022). Nudging healthy food choices: Verifying effectiveness in the cafeteria and considering ethical issues. J. Jpn. Soc. Shokuiku.

[15] Sakaguchi K., Takemi Y., Hayashi F., Koiwai K., Nakamura M. (2021). Effect of workplace dietary intervention on salt intake and sodium-to-potassium ratio of Japanese employees: A quasi-experimental study. J. Occup. Health.

[16] Slapø H.B., Karevold K.I. (2019). Simple eco-labels to nudge customers toward the most environmentally friendly warm dishes: An empirical study in a cafeteria setting. Front. Sustain. Food Syst..

[17] Watanabe A., Fukuda Y. (2016). Effect of dish order on food intake in a buffet lunch among Japanese university students. J. Jpn. Soc. Health Educ. Promot..

[18] Cadario R., Chandon P. (2020). Which healthy eating nudges work best? A meta-analysis of field experiments. Mark. Sci..

[19] Fujita M. (2018). Relation between user’s engagement and motivation in gamification context. J. Inf. Manag..

[20] Asken Inc. Asken. https://www.asken.inc/.

[21] CALOMEAL Inc. CALOMEAL. https://www.calomeal.com/about-calomeal/.

[22] FiNC Technologies Inc. FiNC. https://finc.com/.

[23] Tokyo Tsushin Inc OWN. https://web.own-dot.com/.

[24] YAZIO YAZIO. https://www.yazio.com/en.

[25] Stehr P., Karnowski V., Rossmann C. (2020). The multi-faceted usage patterns of nutrition apps: A survey on the appropriation of nutrition apps among German-speaking users of MyFitnessPal. BMC Med. Inform. Decis. Mak..

[26] Eigen Y., Nishiyama Y., Okoshi T., Nakazawa J. (2019). HealthyStadium: Meal photo SNS with mutual healthiness evaluation for improving users’ eating habits. IPSJ J..

[27] Forman E.M., Manasse S.M., Dallal D.H., Crochiere R.J., Berry M.P., Butryn M.L., Juarascio A.S. (2021). Gender differences in the effect of gamification on weight loss during a daily, neurocognitive training program. Transl. Behav. Med..

[28] Froome H.M., Townson C., Rhodes S., Franco-Arellano B., LeSage A., Savaglio R., Brown J.M., Hughes J., Kapralos B., Arcand J. (2020). The effectiveness of the Foodbot Factory mobile serious game on increasing nutrition knowledge in children. Nutrients.

[29] Gómez-García G., Marín-Marín J.A., Romero-Rodríguez J.M., Ramos Navas-Parejo M., Rodríguez Jiménez C. (2020). Effect of the flipped classroom and gamification methods in the development of a didactic unit on healthy habits and diet in primary education. Nutrients.

[30] Mack I., Reiband N., Etges C., Eichhorn S., Schaeffeler N., Zurstiege G., Gawrilow C., Weimer K., Peeraully R., Teufel M. (2020). The kids obesity prevention program: Cluster randomized controlled trial to evaluate a serious game for the prevention and treatment of childhood obesity. J. Med. Internet Res..

[31] Nah F.F.-H., Eschenbrenner B., Claybaugh C.C., Koob P.B. (2019). Gamification of enterprise systems. Systems.

[32] FoodLog Athl. FoodLog Athl. http://foodlog-athl.org/.

[33] Nakamoto K., Kumazawa K., Karasawa H., Amano S., Yamakata Y., Aizawa K. (2022). Foodlog Athl: Multimedia food recording platform for dietary guidance and food monitoring. Proceedings of the 4th ACM International Conference on Multimedia in Asia (MMAsia ’22).

[34] Ministry of Health, Labour and Welfare (MHLW) (2019). Dietary Reference Intakes for Japanese (2020). https://www.mhlw.go.jp/content/10900000/001150922.pdf.

[35] Ajzen I. (1991). The theory of planned behavior. Organ. Behav. Hum. Decis. Process..

[36] Fishbein M., Ajzen I. (1975). Predicting and understanding consumer behavior: Attitude–behavior correspondence. Understanding Attitudes and Predicting Social Behavior.

[37] McDermott M.S., Oliver M., Svenson A., Simnadis T., Beck E.J., Coltman T., Iverson D., Caputi P., Sharma R. (2015). The theory of planned behaviour and discrete food choices: A systematic review and meta-analysis. Int. J. Behav. Nutr. Phys. Act..

[38] Ajzen I., Kruglanski A.W. (2019). Reasoned action in the service of goal pursuit. Psychol. Rev..

[39] Nakamura S., Inayama T., Harada K., Arao T. (2024). Attitudes, subjective norms, self-efficacy, and behavioral change stages related to adults eating vegetables. Simultaneous multi-population analysis by household income. J. Jpn. Soc. Health Educ. Promot..

[40] Herman C.P., Polivy J. (2005). Normative influences on food intake. Physiol. Behav..

[41] Ajzen I. Attitude Toward the Behavior; Subjective Norm; Intention; Behavior. n.d. https://people.umass.edu/aizen/.

[42] Prochaska J.O., Velicer W.F. (1997). The transtheoretical model of health behavior change. Am. J. Health Promot..

[43] Ministry of Health, Labour and Welfare (MHLW) Behavioral Change Stages Model. https://kennet.mhlw.go.jp/information/information/exercise/s-07-001.

[44] Bandura A. (1977). Self-efficacy: Toward a unifying theory of behavioral change. Psychol. Rev..

[45] Bandura A. (2004). Health promotion by social cognitive means. Health Educ. Behav..

[46] Parkinson J., David P., Rundle-Thiele S. (2017). Self-efficacy or perceived behavioural control: Which influences consumers’ physical activity and healthful eating behaviour maintenance?. J. Consum. Behav..

[47] Schwartz S.H. (1968). Awareness of consequences and the influence of moral norms on interpersonal behavior. Sociometry.

[48] Stern P.C., Dietz T., Abel T.D., Guagnano G., Kalof L. (1999). A value-belief-norm theory of support for social movements: The case of environmentalism. Hum. Ecol. Rev..

[49] Schwartz S.H., Howard J.A., Rushton J.P., Sorrentino R.M. (1981). A normative decision-making model of altruism. Altruism and Helping Behavior.

[50] Ministry of Education, Culture, Sports, Science and Technology (MEXT) (2020). Standard Tables of Food Composition in Japan (8th Rev.). https://www.mext.go.jp/a_menu/syokuhinseibun/mext_01110.html.

[51] Ajzen I. (2011). The theory of planned behaviour: Reactions and reflections. Psychol. Health.

[52] Hu L.-T., Bentler P.M. (1998). Fit indices in covariance structure modeling: Sensitivity to underparameterized model misspecification. Psychol. Methods.

[53] Hu L.-T., Bentler P.M. (1999). Cutoff criteria for fit indexes in covariance structure analysis: Conventional criteria versus new alternatives. Struct. Equ. Model..

[54] Hayes A.F. (2013). Introduction to Mediation, Moderation, and Conditional Process Analysis: A Regression-Based Approach.

[55] Hayes A.F. (2024). The PROCESS macro for SPSS, SAS, and R. https://processmacro.org/index.html.

[56] Bearden W.O., Sharma S., Teel J.E. (1982). Sample size effects on chi square and other statistics used in evaluating causal models. J. Mark. Res..

[57] Bollen K.A. (1990). Overall fit in covariance structure models: Two types of sample size effects. Psychol. Bull..

[58] Iacobucci D. (2010). Structural equation modeling: Fit indices, sample size, and advanced topics. J. Consum. Psychol..

[59] Godin G., Kok G. (1996). The theory of planned behavior: A review of its applications to health-related behaviors. Am. J. Health Promot..

[60] McEachan R.R.C., Conner M., Taylor N.J., Lawton R.J. (2011). Prospective prediction of health-related behaviours with the Theory of Planned Behaviour: A meta-analysis. Health Psychol. Rev..

[61] Fila S.A., Smith C. (2006). Applying the Theory of Planned Behavior to healthy eating behaviors in urban Native American youth. Int. J. Behav. Nutr. Phys. Act..

[62] Dolgopolova I., Li B., Pirhonen H., Roosen J. (2022). The effect of attribute framing on consumers’ attitudes and intentions toward food: A Meta-analysis. Bio-Based Appl. Econ..

[63] Ministry of Internal Affairs and Communications (MIAC) (2020). Trends and outlook for household composition. https://www.mhlw.go.jp/content/12600000/001334405.pdf.

[64] Yoshida T., Watanabe D., Nakagata T., Yamada Y., Kurotani K., Sawada N., Tanaka K., Okabayashi M., Shimada H., Takimoto H. (2021). Prevalence of frailty and its related factors in community-dwelling middle-aged and elderly adults in Settsu and Hannan cities in Osaka Prefecture. Jpn. J. Public Health.

