# Supplementary material for: Developing an Empirical Theory of Planned Behavior Model of Healthy Dietary Choice and Evaluating Gamified Feedback Among Japanese Young Adults
Source: Nutrients. 2026 Feb 20;18(4):686. doi: 10.3390/nu18040686 (PMC12943355; doi:10.3390/nu18040686)
Supplement: Supplementary file 1 [file nutrients-18-00686-s001.zip › Supplementary_Table_S1r.pdf]

Table S1. Comprehensive evaluation of components in the dietary choice behavior model.

				**Person making dietary choices (PDC)**				**Cognitive (CO)**				**Attitude toward the behavior (ATB)**				**Subjective norm (SN)**				**Self-efficacy (SE)**			
		n	%	Mean (%)	SD	SE	***p***	Mean (%)	SD	SE	***p***	Mean (%)	SD	SE	***p***	Mean (%)	SD	SE	***p***	Mean (%)	SD	SE	***p***
Overall (*N*)		188	100	73.1	25.2	1.8		66.4	21.9	1.6		66.2	15.9	1.2		71.3	16.6	1.2		54.1	17.5	1.3	
Gender	Male	138	73.4	72.3	25.0	2.1	1.000[b]	68.6	21.1	1.8	0.130	64.9	15.6	1.3	0.261	72.1	17.3	1.5	0.515	54.6	17.5	1.5	1.000
	Female	48	25.5	75.4	26.2	3.8		61.1	22.6	3.3		70.0	16.6	2.4		69.2	14.8	2.1		52.1	17.3	2.5	
	N/A[a]	2	1.1	70.0	14.1	10.0		42.0	35.4	25.0		65.0	7.1	5.0		70.0	4.2	3.0		72.5	24.7	17.5	
Age	18～29	157	83.5	71.8	25.3	2.0	0.120	66.8	21.4	1.7	0.709	65.3	16.7	1.3	0.111	72.1	16.3	1.3	0.234	54.3	17.8	1.4	0.743
	30s	31	16.5	79.4	23.9	4.3		64.5	24.6	4.4		71.0	16.4	2.9		67.5	17.8	3.2		53.4	16.5	3.0	
Students' univ. year (Student: n=153/188)	1st	2	1.1	70.0	42.4	30.0		75.0	11.3	8.0		65.0	7.1	5.0		63.5	4.9	3.5		60.0	7.1	5.0	
	2nd	109	58.0	70.1	24.9	2.4		65.6	20.8	2.0		63.9	15.8	1.5		73.8	16.0	1.5		56.3	18.6	1.8	
	3rd	7	3.7	62.9	26.9	10.2		64.4	31.1	11.7		65.7	12.7	4.8		63.9	18.9	7.1		42.9	9.9	3.7	
	4th	16	8.5	77.5	28.2	7.0		65.7	14.2	3.5		68.1	16.4	4.1		75.0	14.5	3.6		53.4	13.1	3.3	
	Graduate school	19	10.1	72.6	25.1	5.8		67.5	26.9	6.2		63.2	13.8	3.2		67.4	12.0	2.8		52.6	14.7	3.4	
Living situation	With family/partner	82	43.6	61.9	22.8	2.5	<0.001	68.7	21.7	2.4	0.130	64.6	15.6	1.7	0.137	71.7	15.7	1.7	0.894	53.8	16.1	1.8	0.964
	Alone	106	56.4	81.7	23.6	2.3		64.6	21.9	2.1		67.5	16.2	1.6		71.0	17.3	1.7		54.4	18.6	1.8	
Usage of diet apps	Always	7	3.7	80.0	20.0	7.5		54.7	25.1	9.5		64.3	25.1	9.5		72.4	12.5	4.7		58.6	14.0	5.3	
	Occasio-nally	7	3.7	82.9	24.3	9.2		52.3	31.1	11.7		71.4	13.5	5.1		74.3	15.6	5.9		47.9	9.9	3.7	
	Rarely	12	6.4	66.7	24.6	7.1		72.2	25.9	7.5		69.2	16.2	4.7		73.4	15.9	4.6		54.2	17.2	4.9	
	No use	162	86.2	72.8	25.5	2.0		67.1	20.8	1.6		65.9	15.6	1.2		71.0	17.0	1.3		54.2	18.0	1.4	
Comparison of predictors across the stages of dietary behavior attitude.																							
1. I'm not interested in my diet and have no intention of improving it.		8	4.3	52.5	28.1	9.9		68.7	16.5	5.8		36.3	10.6	3.8		68.4	12.7	4.5		43.7	12.5	4.4	
2. I'm interested in my diet, but I think it's fine the way it is even if it's a little unbalanced.		51	27.1	69.0	24.4	3.4		65.4	19.4	2.7		54.1	9.0	1.3		68.2	14.5	2.0		55.9	13.7	1.9	
3. I'm interested in my diet, so I'd like to improve it if the opportunity arises.		99	52.7	72.3	24.4	2.4		66.1	23.3	2.3		68.3	10.3	1.0		71.0	18.0	1.8		51.5	18.7	1.9	
4. I'm interested in my diet and have been trying to improve it for less than 6 months.		17	9.0	88.2	21.3	5.2	0.011 (1-4)[c]<br>0.032 (2-4)	66.8	21.2	5.1		84.7	6.2	1.5		80.1	13.6	3.3		54.4	15.8	3.8	
5. I'm already practicing a better diet and continuing it.		13	6.9	87.7	23.8	6.6	0.019 (1-5)	70.5	25.5	7.1		92.3	12.4	3.4		75.9	16.1	4.5		73.8	12.4	3.5	0.001 (1-5)<br>0.020 (2-5)<br><0.001 (3-5)<br>0.021 (4-5)

to be continued

Continued from the previous page

				Intention (INT)				Behavior (BE)				Awareness of consequences (AC)				Ascription of responsibility (AR)				Personal norm (PN)			
		n	%	Mean (%)	SD	SE	*p*	Mean (%)	SD	SE	*p*	Mean (%)	SD	SE	*p*	Mean (%)	SD	SE	*p*	Mean (%)	SD	SE	*p*
Overall (*N*)		188	100	76.1	14.8	1.1		54.8	17.3	1.3		87.4	14.0	1.0		76.2	16.2	1.2		64.8	17.3	1.3	
Gender	Male	138	73.4	75.1	15.4	1.3	0.339	55.2	18.3	1.6	1.000	86.9	14.6	1.2	1.000	76.6	16.2	1.4	1.000	63.5	17.0	1.4	0.233
	Female	48	25.5	78.6	12.6	1.8		53.1	14.2	2.0		88.2	12.3	1.8		75.1	16.7	2.4		68.4	17.7	2.5	
	N/A	2	1.1	83.0	14.1	10.0		70.0	0.0	0.0		100.0	0.0	0.0		73.0	0.0	0.0		70.0	21.2	15.0	
Age	18～29	157	83.5	75.5	15.0	1.2	0.198	54.6	17.8	1.4	0.784	86.7	14.4	1.1	0.136	75.2	16.4	1.3	0.033	64.9	17.2	1.4	0.877
	30s	31	16.5	79.1	13.5	2.4		56.0	14.6	2.6		90.9	11.4	2.0		81.6	14.4	2.6		64.3	17.7	3.2	
Students' univ. year (Student: n=153/188)	1st	2	1.1	80.0	0.0	0.0		50.0	7.1	5.0		80.0	0.0	0.0		86.5	19.1	13.5		62.5	10.6	7.5	
	2nd	109	58.0	75.7	15.5	1.5		55.8	18.1	1.7		86.1	14.7	1.4		74.4	15.5	1.5		65.8	17.8	1.7	
	3rd	7	3.7	72.4	18.6	7.0		41.4	18.6	7.0		82.9	17.1	6.5		63.6	23.1	8.7		56.4	23.6	8.9	
	4th	16	8.5	81.3	12.0	3.0		56.6	14.9	3.7		91.3	9.9	2.5		79.9	16.8	4.2		68.4	13.7	3.4	
	Graduate school	19	10.1	73.4	11.3	2.6		53.7	14.1	3.2		84.6	15.6	3.6		78.3	12.6	2.9		64.2	12.2	2.8	
Living situation	With family/partner	82	43.6	76.2	12.9	1.4	0.838	56.4	15.5	1.7	0.231	86.8	14.7	1.6	0.754	72.2	15.6	1.7	0.001	64.8	14.9	1.6	0.825
	Alone	106	56.4	76.0	16.1	1.6		53.6	18.6	1.8		87.8	13.6	1.3		79.3	16.1	1.6		64.9	19.0	1.8	
Usage of diet apps	Always	7	3.7	73.3	14.9	5.6		58.6	12.8	4.8		85.7	16.2	6.1		79.0	22.6	8.5		70.7	16.7	6.3	
	Occasionally	7	3.7	75.1	15.3	5.8		52.1	10.3	3.9		82.0	21.3	8.0		76.1	25.6	9.7		67.9	13.8	5.2	
	Rarely	12	6.4	76.7	16.4	4.7		53.8	17.2	5.0		91.1	11.8	3.4		73.3	16.3	4.7		71.7	14.2	4.1	
	No use	162	86.2	76.2	14.7	1.1		54.9	17.8	1.4		87.4	13.8	1.1		76.3	15.6	1.2		63.9	17.6	1.4	
Comparison of predictors across the stages of dietary behavior attitude.																							
1. I'm not interested in my diet and have no intention of improving it.		8	4.3	57.5	12.7	4.5		42.5	16.0	5.7		86.8	15.4	5.5		80.6	17.2	6.1		45.6	15.7	5.5	
2. I'm interested in my diet, but I think it's fine the way it is even if it's a little unbalanced.		51	27.1	71.2	12.1	1.7		58.0	16.1	2.3		83.0	14.6	2.0		71.6	13.1	1.8		59.6	15.2	2.1	
3. I'm interested in my diet, so I'd like to improve it if the opportunity arises.		99	52.7	77.6	14.2	1.4	0.006 (1-3) 0.056 (2-3)	51.6	15.9	1.6		87.4	14.2	1.4		75.8	16.6	1.7		67.0	16.9	1.7	0.011 (1-3)
4. I'm interested in my diet and have been trying to improve it for less than 6 months.		17	9.0	82.4	13.1	3.2	0.003 (1-4) 0.056 (2-4)	55.3	17.4	4.2		95.7	7.0	1.7	0.004 (2-4)	85.5	17.4	4.2	0.007 (2-4)	72.7	16.1	3.9	0.008 (1-4)
5. I'm already practicing a better diet and continuing it.		13	6.9	87.2	16.9	4.7	<0.001 (1-5) 0.004 (2-5)	73.8	18.8	5.2	0.004 (1-5) 0.002 (3-5)	93.8	10.3	2.8	0.051 (2-5)	82.5	16.9	4.7		70.4	18.5	5.1	0.026 (1-5)

[a] N/A indicates "no answer."

[b] *P* value between male and female difference was indicated.

[c] Values in parentheses indicate which stages were compared.
