# Supplementary material for: Developing an Empirical Theory of Planned Behavior Model of Healthy Dietary Choice and Evaluating Gamified Feedback Among Japanese Young Adults
Source: Nutrients. 2026 Feb 20;18(4):686. doi: 10.3390/nu18040686 (PMC12943355; doi:10.3390/nu18040686)
Supplement: Supplementary file 1 [file nutrients-18-00686-s001.zip › Supplementary_Table_S2r.pdf]

Table S2. Changes in sample size during the FoodLog Athl experiment (N = 183).

**Number of participants:**	**(1) were distributed FoodLog Athl**	**(2) downloaded app**	**(2)/(1)**	**(3) recorded meals**	**(3)/(2)**	**(4) responded to post-survey questionnaire**	**(4)/(1)**	**(5) matched between post-survey and meals recording**	**(5)/(4)**
Ratings	*n*/*N*	*n*	%	*n*	%	*n*	%	*n*	%
Star-	92/183	35/92	38	21/35	60	25[a]/92	27	18/25	72
Non-	91/183	27/91	30	20/27	74	16/91	18	13/16	81

Notes: n/N indicates the number of participants at each stage relative to the number in the preceding stage.

[a] Includes participants who responded the post-survey questionnaire without finishing the meal record task.
